# First human case report of bloodstream infection caused by *Kocuria indica* in a patient with chronic idiopathic intestinal pseudo-obstruction

**DOI:** 10.1128/asmcr.00066-24

**Published:** 2025-04-23

**Authors:** Daisuke Kitagawa, Shuhei Kogata, Kengo Nakahata, Shohei Kishida, Koki Kimura, Katsuji Yamauchi, Takeo Yonekura, Taito Kitano, Takehito Kasamatsu, Naoyuki Shiraishi, Soma Suzuki, Ayu Yamamoto, Ritsuki Uejima, Yuki Suzuki, Akiyo Nakano, Ryuichi Nakano, Hisakazu Yano, Koichi Maeda, Fumihiko Nakamura

**Affiliations:** 1 Department of Laboratory Medicine, Nara Prefecture General Medical Center 274917, Nara, Japan; 2 Department of Microbiology and Infectious Diseases, Nara Medical University 543244, Kashihara, Japan; 3 Department of Pediatric Surgery, Nara Prefecture General Medical Center 36927, Nara, Japan; 4 Department of Pediatric Surgery, Osaka University Graduate School of Medicine 595209 , Suita, Japan; 5 Department of Pediatrics, Nara Prefecture General Medical Center 36927, Nara, Japan; 6 Department of Infectious Diseases, Nara Prefecture General Medical Center 36927, Nara, Japan; Vanderbilt University Medical Center, Nashville, Tennessee, USA

**Keywords:** *Kocuria indica*, bloodstream infection, pneumonia, matrix-assisted laser desorption-ionization time-of-flight mass spectrometry, 16S ribosomal RNA gene sequencing

## Abstract

**Background:**

*Kocuria* spp. are aerobic Gram-positive cocci and opportunistic pathogens. Although infections caused by *Kocuria* spp. have been reported, to the best of our knowledge, human infections caused by *Kocuria indica* have not been reported. Here, we report a case of pneumonia and bloodstream infection caused by *K. indica*.

**Case Summary:**

This is the first reported case of human *K. indica* infection. A 19-year-old female with a history of chronic idiopathic intestinal pseudo-obstruction presented with fever, cough, and malaise. Blood cultures revealed clusters of Gram-positive cocci. The organism was initially identified as *Kocuria rhizophila* using the VITEK MS system but was confirmed as *K. indica* using 16S rRNA gene sequencing.

**Conclusion:**

Although *K. indica* is a rare pathogen, it should be considered in cases of positive blood cultures. Genetic analysis, including 16S rRNA gene sequencing, is essential for the identification of this organism.

## INTRODUCTION

The *Kocuria* genus includes Gram-positive cocci belonging to the *Micrococcaceae* family, Actinomycota order, and actinomycetes, and exhibits a catalase-positive, coagulase-negative, clustered shape (1). These ubiquitous bacteria are found in diverse environments, including soil, fresh and marine water, desert sand, plant rhizosphere, fermented foods, and various animal sources (2). They have been isolated from the normal human skin microbiota, oropharynx, and mucous membranes, as well as from hospital surface and air samples (2, 3).

*Kocuria* consists of 18 species, some of which are opportunistic human pathogens that primarily affect immunocompromised or severely ill patients (4). Traditionally considered nonpathogenic commensals, *Kocuria* species have increasingly been recognized as clinically significant over the past two decades. *Kocuria* infection cases more than doubled between 2000–2010 and 2011–2020, owing to improved detection methods, increased use of long-term intravascular devices, and increased population of immunocompromised individuals (5, 6).

*Kocuria* species can cause opportunistic infections, including bacteremia, endocarditis, peritonitis, cholecystitis, catheter-associated urinary tract infections, and skin and soft tissue infections, with catheter-related bloodstream infection, particularly in patients with central venous catheters, being the most frequent (7). Risk factors for *Kocuria* infections include malignancies, immunosuppression, chronic kidney disease requiring peritoneal dialysis, metabolic disorders, and presence of indwelling medical devices (8). Among the various species, *Kocuria kristinae* is most common in clinical specimens, followed by *Kocuria rhizophila* and *Kocuria varians* (9).

*Kocuria indica*, a rare organism, first isolated from marine sediments off the Indian coast in 2014 (10), is not often detected in human infections, and only one case from skin is retrievable from PubMed (11). *K. indica* infection remains poorly understood due to its rarity and the historical challenges in accurate species-level identification of *Kocuria* isolates before the widespread implementation of molecular methods. Here, we report, to the best of our knowledge, the first case of *K. indica* bloodstream infection, contributing to the expanding knowledge of the clinical spectrum and pathogenic potential of this uncommon species.

## CASE PRESENTATION

**Fig 1:**
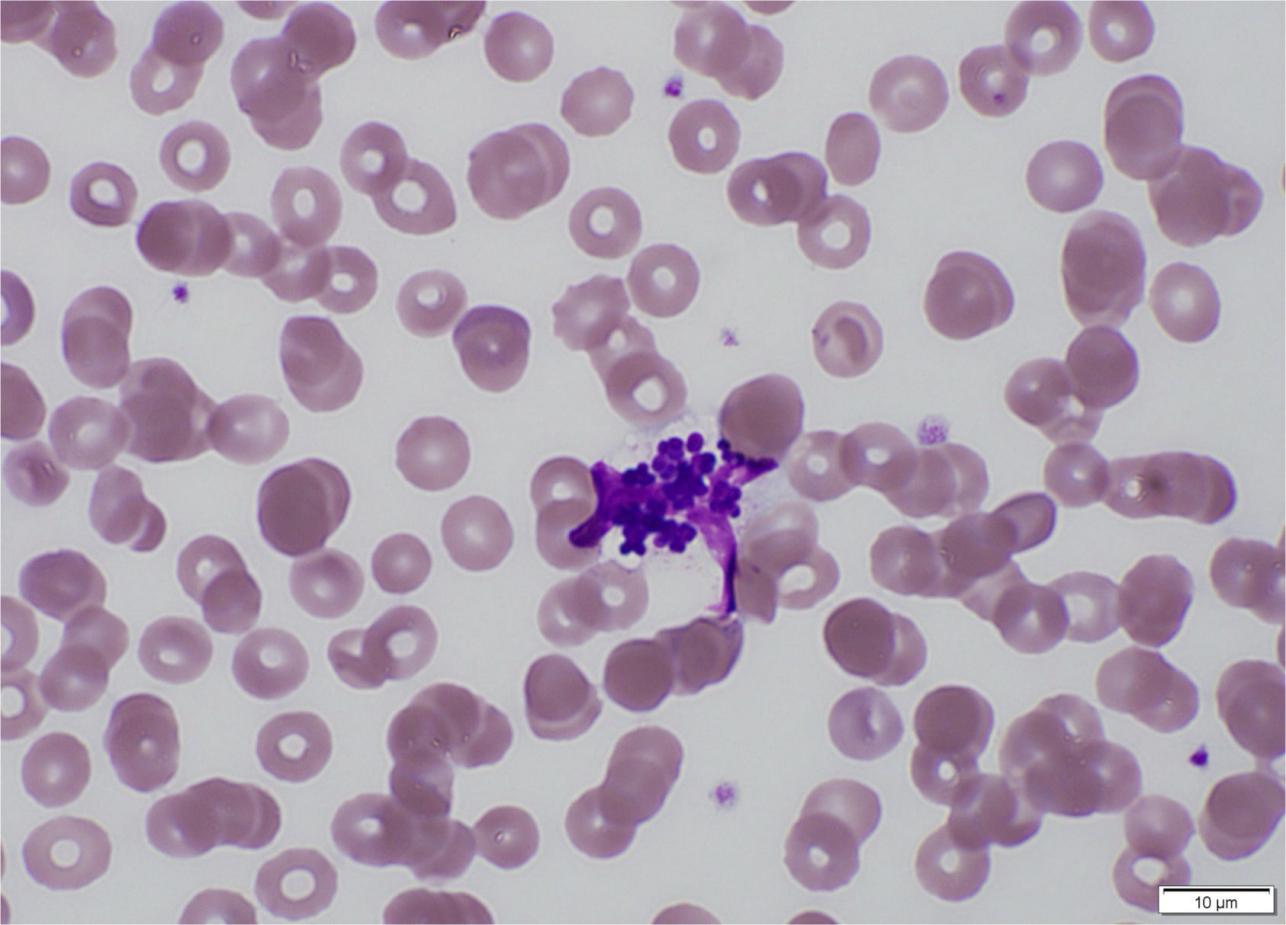
Giemsa-stained image of blood taken from the central venous catheter with port (on admission). Cocci with neutrophil phagocytosis can be seen.

A 19-year-old female, who was being treated at Nara Prefecture General Medical Center for chronic idiopathic intestinal pseudo-obstruction (CIIP), presented to the Department of Pediatric Surgery with complaints of fever, cough, and malaise. Her coughing symptoms appeared gradually over 4 days, and a day before admission in our hospital, she was diagnosed with a respiratory tract rhinovirus infection using FilmArray Respiratory Panel 2.1 (FilmArray; Biofire, Salt Lake, UT, USA) in the same department. At arrival, her temperature was 39.2°C, heart rate was 152 /min, and oxygen saturation was 88%. Results of admission laboratory tests are shown in Table 1. Blood smears (Giemsa-stained) from a central venous catheter with a port demonstrated *Staphylococcus*-like bacteria phagocytosed by leukocytes (Fig. 1). Chest X-ray showed ground-glass opacities on both lower lung fields (but predominant on the left side). Sputum culture could not be obtained. The patient had undergone extensive resection of the small intestine and left hemicolon for CIIP and jejunostomy, gastrostomy, and colostomy 7 years earlier, had a central venous catheter placed 2 years prior, and required daily central venous nutritional management. The patient was admitted for advanced management of a bloodstream infection (BSI) and pneumonia. The definition of central line-associated bloodstream infection (CLABSI) according to the National Healthcare Safety Network was not met given the presence of pneumonia (12).

**TABLE 1:** Main laboratory results at outpatient clinic^*a*^

Parameter	Value
WBC (33–86 × 10^2^/µL)	82 × 10^2^/µL
Neu (42.0%–75.6 %)	88.1%
Lym (17.4%–48.2 %)	5.4%
Mon (3.4%–9.0 %)	3.8%
Eosino (0.4%–8.6 %)	1.3%
Baso (0.2%–1.4 %)	0.5%
Toxic granulation	+^*b*^
Doehle body	+
Bacterial phagocytosis	+
RBC (386–492 × 10^4^/µL)	318 × 10^4^/µL
Hb (11.6–14.8 g/dL)	9.9 g/dL
Hct (35.1%–44.4%)	27.9%
MCV (83.6–98.2 fL)	87.8 fL
MCH (27.5–33.2 pg)	31.1 pg
MCHC (31.7–35.3 g/dL)	35.4 g/dL
Plt (15.8–34.8 × 10^4^/µL)	12.5 × 10^4^ /µL
Na (138–145 mmol/L)	139 mmol/L
K (3.6–4.8 mmol/L)	4.4 mmol/L
Cl (101–108 mmol/L)	106 mmol/L
AST (13–30 IU/L)	44 IU/L
ALT (7–23 IU/L)	52 IU/L
LD (124–222 IU/L)	354 IU/L
ALP (38–113 IU/L)	262 IU/L
γ-GTP (9–32 IU/L)	33 IU/L
IP (2.7–4.6 mg/dL)	2.6 mg/dL
CRP (< 0.14 mg/dL)	5.5 mg/dL
BUN (8–20 mg/dL)	17.1 mg/dL
CRE (0.46–0.79 mg/dL)	0.58 mg/dL
Procalcitonin (<0.05 ng/mL)	7.46 ng/mL

^*a*^ Reference ranges are presented with parentheses.

^*b*^ +, observed on blood test smear findings.

**Fig 2:**
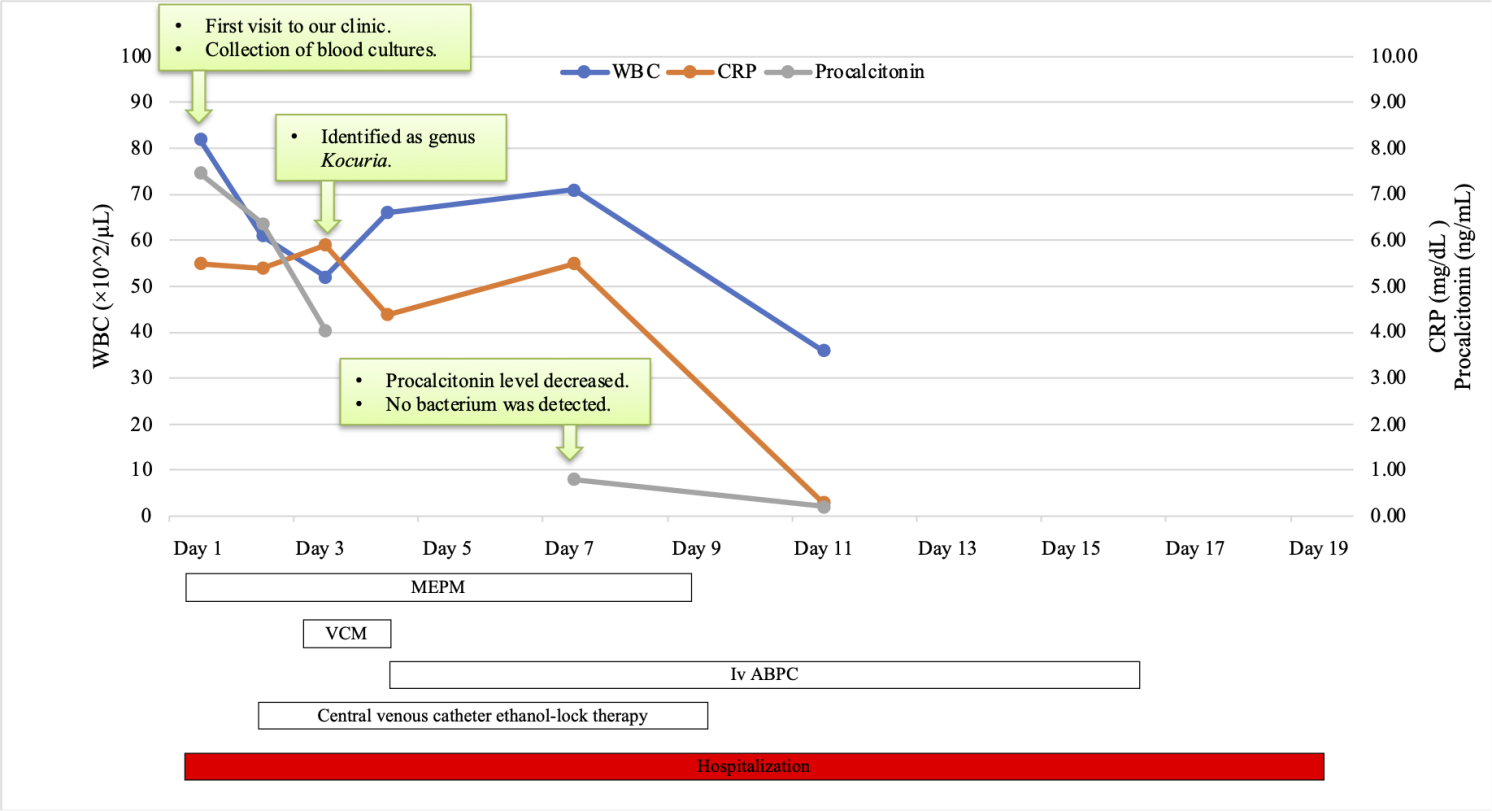
White blood cell count and levels of C-reactive protein (CRP) and procalcitonin based on clinical course during hospitalization.

After two sets of blood cultures from two different lumens of the central line were obtained, intravenous meropenem (120 mg/kg/day) was started on the first day of admission because of previous history of recurrent infections with extended-spectrum beta-lactamase-producing Enterobacterales. On the second day of admission, Gram-positive cocci were detected in three of the four blood cultures, and intravenous vancomycin (60 mg/kg/day) was added to the treatment. The central venous catheter was not removed due to difficult venous access, and ethanol lock therapy was administered for 8 days, starting day 2 of admission. On the third day of admission, the organism was identified as *Kocuria* spp. Based on the results of antimicrobial susceptibility testing (Table 2), the patient was switched from vancomycin to intravenous ampicillin (200 mg/kg/day). Although there are no breakpoints for *Kocuria* spp., ampicillin was selected considering the minimum inhibitory concentrations and to treat the infection with a narrow-spectrum antimicrobial. Meropenem was continued for 8 days because of concerns regarding congestive enteritis. The patient completed 14 days of antimicrobial treatment after the first negative blood culture. She was discharged on the 18th day of hospitalization after confirmation of negative blood cultures on the third and seventh day of hospitalization, with a decrease in C-reactive protein (CRP) and procalcitonin levels and improvement in her respiratory status and fever (Fig. 2). The patient underwent replacement of the central venous catheter due to a break in the catheter connection 1 month after discharge.

**TABLE 2:** Antimicrobial susceptibility profiles of *Kocuria* isolate using the dry plate (Eiken Chemical)

Antimicrobial agent	MIC (μg/mL)
Penicillin	≤0.12
Ampicillin	≤0.12
Ampicillin/sulbactam	≤2
Piperacillin	≤2
Piperacillin/tazobactam	≤2
Cefazolin	≤2
Cefmetazole	≤1
Flomoxef	≤1
Ceftriaxone	≤1
Ceftazidime	≤1
Cefepime	≤1
Imipenem	≤0.25
Meropenem	≤0.25
Levofloxacin	4
Moxifloxacin	2
Gentamicin	≤1
Arbekacin	≤1
Vancomycin	≤0.5
Teicoplanin	≤0.5
Daptomycin	≤0.25
Linezolid	1
Erythromycin	≤0.25
Clindamycin	2
Minocycline	≤0.25
Fosfomycin	32
Rifampicin	≤0.5
Sulfamethoxazole/trimethoprim	>40

**Fig 3:**
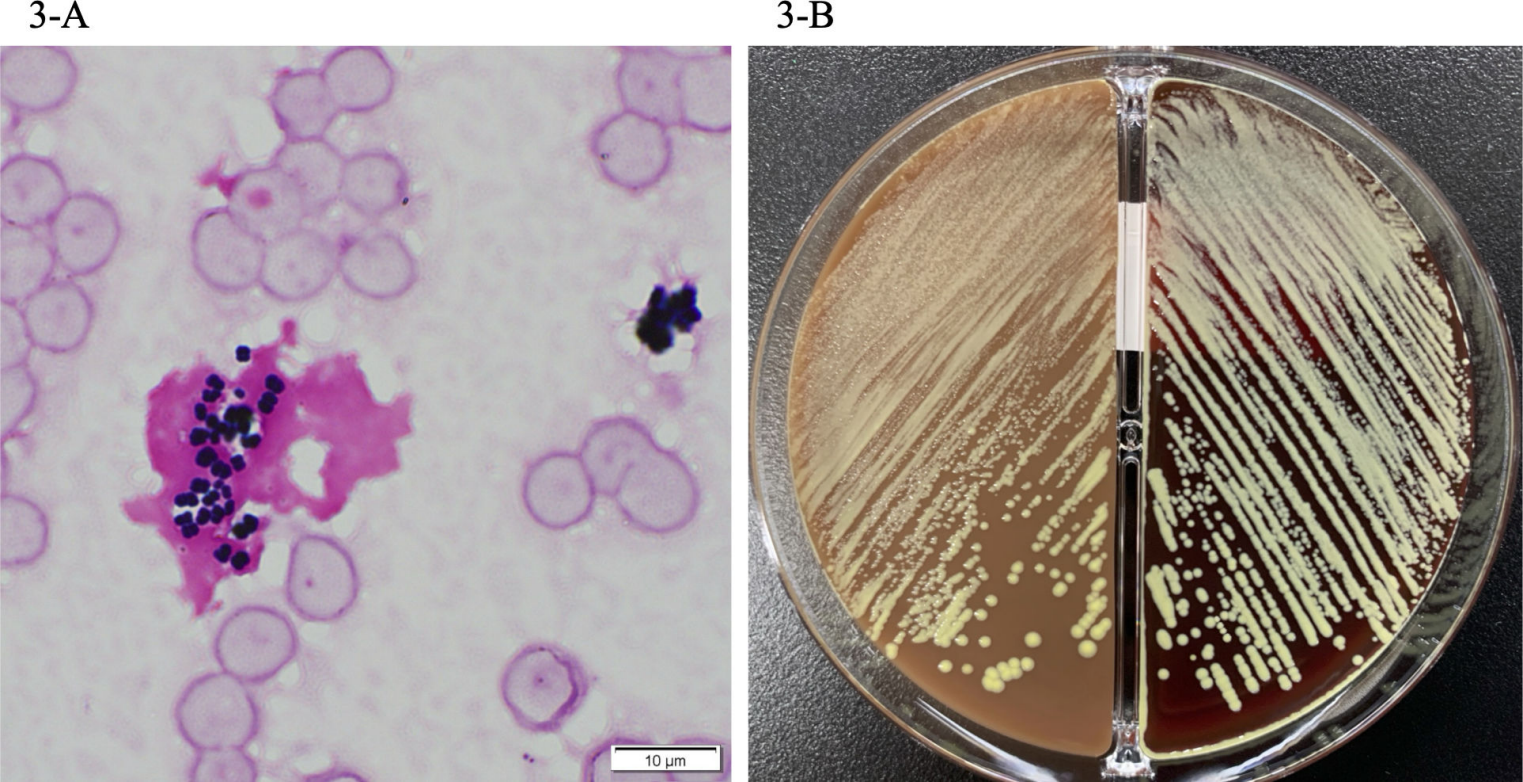
Phenotypic features of *Kocuria indica*. (**A**) *Kocuria indica* appeared as clustered Gram-positive cocci. (**B**) On blood agar and chocolate agar media, *K. indica* isolates appeared as yellow colonies.

**Fig 4:**
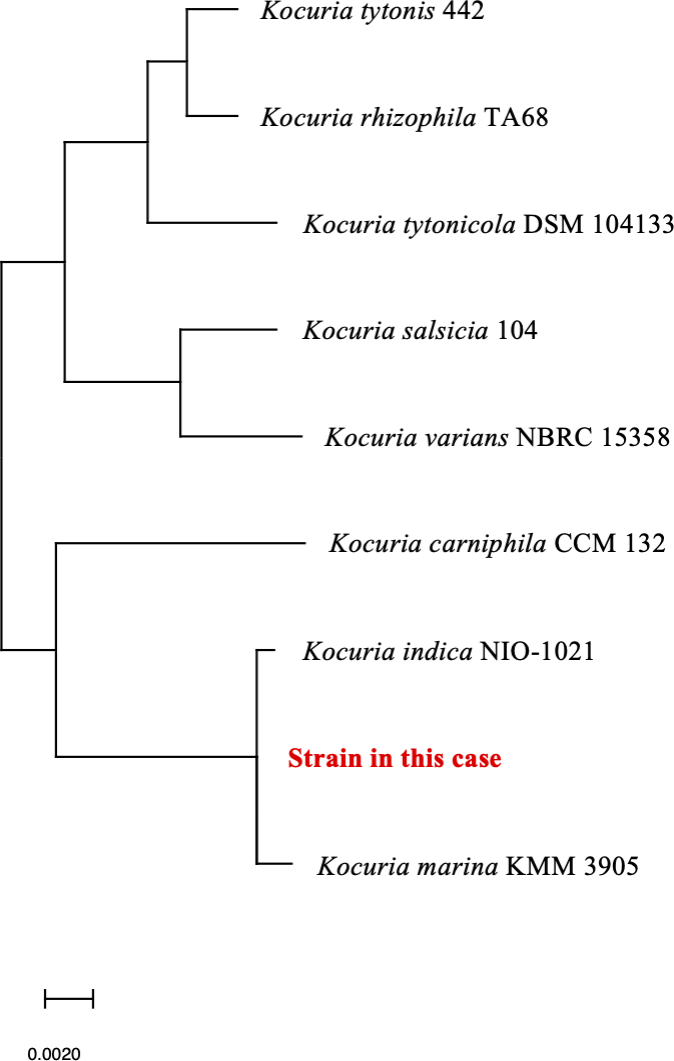
Results of phylogenetic analysis using strains with high homology. The closest homology was determined to be with *Kocuria indica* NIO-1021.

Two sets of blood cultures were collected in standard aerobic (SA) and standard anaerobic (SN) bottles (bioMérieux, Marcyl’Etoile, France) on visit to the emergency department. All the cultures (4/4) were found positive using the BacT/Alert 3D system (bioMérieux) after 11 h of incubation. Gram staining showed numerous Gram-positive cocci in a background of numerous polymorphonuclear leukocytes and red blood cells (Fig. 3A). The FilmArray BCID-2 (bioMérieux), a multiparameter PCR test, was negative for all panel organisms. The cultures formed yellow colonies without hemolysis on blood agar medium (Becton Dickinson, Franklin Lakes, NJ, USA) or chocolate agar medium (Becton Dickinson) (Fig. 3B). The organism was identified as *Kocuria rhizophila* using mass spectrometry (VITEK MS, bioMérieux). However, 16S ribosomal RNA (rRNA) gene sequencing using universal primers, 27F (5′-AGTTTGATCMTGGCTCAG-3′) and 1492R (5′-TACGGGYTACCTTGTTACGACTT-3′) confirmed that *K. rhizophila* is 99.72% (1,400/1,404 bp) identical to the *K. indica* NIO-1021(T) strain (GenBank accession number FXAC01000036) and 99.64% (1399/1404 bp) identical to *Kocuria marina* KMM 3905(T) strain (GenBank accession number AY211385) (13). The identification results were obtained from the website https://www.ezbiocloud.net/ (database update: 23 August 2023) (14). The phylogenetic tree based on the EzBioCloud Database using 16S rRNA gene sequencing of these strains showed the highest homology to *K. indica* (Fig. 4).

The minimum inhibitory concentrations of 27 antimicrobial agents were determined using a DryPlate 42 (Eiken Chemical Co., Ltd., Tokyo, Japan) (Table 2). The dry plate is a microplate in which antimicrobials are dispensed at various dilutions into wells and allowed to dry. Using a bacterial colony grown on sheep blood agar medium for 24 h, the turbidity of the suspension was adjusted with sterile saline to be the same as the McFarland standard turbidity of 1.0. Thereafter, 0.025 mL of the adjusted inoculum was added to 12 mL of Muller–Hinton Bouillon “Eiken” with 1.0 mL of Strepto Hemo-Supplement “Eiken” and mixed evenly to prepare the inoculum solution. This inoculum (0.1 mL; approximately 5 × 10^4^ colony-forming units (CFU)/well) was added to each well of the dry plate, which was then incubated under aerobic conditions at 35°C for 24–48 h for determining the antimicrobial susceptibility.

## DISCUSSION

*K. indica* is a catalase-positive, coagulase-negative, Gram-positive, non-motile coccus belonging to the *Kocuria* genus (11). It was first isolated from the sediments of Cholao Island, India, by Dastager et al. in 2014 (10). Common *Kocuria* spp. are part of the normal human flora that inhabit mucous membranes, such as those in the skin and oral cavity, and are isolated from the environment (15). Only one case of *K. indica* infection has been reported from human skin, without any description of the signs of infection (3). For other *Kocuria* species, clinical presentations vary from bacteremia, skin, and soft tissue infections (SSTIs), endophthalmitis, infective endocarditis, and peritonitis, with the most frequently identified species being *Kocuria kristinae*, which has a low overall mortality rate (15). A predisposition to *Kocuria* spp. is associated with serious underlying diseases and long-term catheter retention (15). The most common medical history among infected patients is malignancy, with cancer chemotherapy increasing the risk of SSTI due to *Kocuria* spp. (16). It has also been reported in chronically ill patients with long-term indwelling catheters who require intravenous nutrition (17). In the present case, the patient required intravenous nutrition because of CIIP and had a long-term indwelling catheter. Malnutrition, chronic intestinal comorbidities, and long-term use of central venous catheters are associated with increased risk of opportunistic infections in patients with bowel obstruction (18, 19). Whether the presence of bacteria in Giemsa-stained specimens of blood before culturing indicates a high bacterial burden needs further investigation. If a patient shows signs of infection under these circumstances, care should be taken to identify BSI at an early stage, including obtaining blood cultures and changing the central venous catheter as needed. A possibility that the patient’s BSI was secondary to pneumonia could not be ruled out, although BSI secondary to pneumonia caused by a commensal is rare, unless the patient is severely immunocompromised (20).

Genetic identification methods have enabled the detection of new and rare bacteria in clinical samples. *K. indica* was identified using 16S rRNA gene sequencing, presumably because it is not included in the databases of commercial identification systems (VITEK MS). Comparative analysis of 16S rRNA gene sequences revealed high homology between *K. indica* and *K. marina*. The two species also form a monophyletic group and are characterized by qualitatively similar cellular fatty acid profiles (3, 7). A phylogenetic tree was created using these two species, which were also highly homologous in the present study, and the bacterium was identified as *K. indica*. Whole-genome sequencing is extremely useful for accurate identification of bacteria. However, its implementation in Japanese hospital laboratories remains challenging due to high costs and technical requirements. In the future, as whole-genome sequencing becomes more widely adopted in clinical laboratories in Japan, it will enable the identification of rare bacterial species and provide deeper insights into the epidemiological backgrounds of various bacteria.

Currently, no standardized antimicrobial susceptibility breakpoints are established for *Kocuria* spp., and delays in diagnosis and treatment have been noted (15). Many strains of *Kocuria* spp. are resistant to penicillin and ampicillin but are susceptible to vancomycin, linezolid, tetracycline, cephalosporins, and amoxicillin-clavulanic acid (15). Vancomycin is an option for empirical therapy following the identification of *Kocuria* spp. with pending antimicrobial susceptibility results (21). The patient’s clinical and laboratory parameters improved after ampicillin treatment. The results of this treatment provide evidence that *K. indica* can cause human infections, including BSI, and contribute to the patient’s condition.

In summary, we report the first human case of BSI caused by *K. indica*. This rare microorganism is difficult to identify using conventional methods. Therefore, methods, such as 16S rRNA gene sequencing, are required for members of the *Kocuria* genus isolated from patients with severe underlying diseases or central venous catheters. This report contributes to further understanding of the clinical impact of *Kocuria* spp. and its subspecies.
